# Genetic landscape of uveal melanoma in Southeast Asia: high 1q gains and unique patterns of metastasis risk

**DOI:** 10.1186/s40662-025-00430-8

**Published:** 2025-04-16

**Authors:** Chuanfei Chen, Mona Meng Wang, Alvin Soon Tiong Lim, Evelyn Yee Hsieh Heng, Sim Leng Tien, Sunny Yu Shen, Gavin Siew Wei Tan, Jason Yongsheng Chan, Anita Sook Yee Chan

**Affiliations:** 1 Cytogenetics Laboratory, Department of Haematology, Singapore General Hospital, Singapore, Singapore; 2 Singapore Eye Research Institute, Singapore, Singapore; 3 Institute of Biomedicine, Faculty of Medicine, University of Turku, Turku, Finland; 4 Singapore National Eye Center, Singapore, Singapore; 5 Ophthalmology & Visual Sciences Academic Clinical Program, Duke-NUS Medical School, Singapore, Singapore; 6 Division of Medical Oncology, National Cancer Center Singapore, Singapore, Singapore

**Keywords:** Chromosomal aberrations, SNP array, Risk stratification, Prediction, Metastasis, Uveal melanoma

## Abstract

**Purpose:**

This retrospective cohort study aims to investigate chromosomal aberrations in Southeast Asian (SEA) uveal melanoma (UM) patients, evaluate their impact on clinical outcomes, and compare findings with the TCGA-Uveal Melanoma (TCGA-UM) dataset to explore potential genetic differences.

**Methods:**

Formalin-fixed paraffin-embedded (FFPE) tumour samples from 20 UM patients diagnosed between 2004 and 2018 were initially analysed using the OncoScan™ CNV Array to detect chromosomal aberrations, with 14 samples yielding valid results for cytogenetic analysis. BAP1 immunohistochemistry was performed on all 20 samples to assess BAP1 protein expression using automated immunostaining techniques validated in the Clinical Pathology Laboratory of the Singapore General Hospital. Clinical data were retrospectively reviewed, and chromosomal aberration frequencies were compared with the TCGA-UM dataset.

**Results:**

A total of 78 chromosomal gains, 48 losses, and two cases of copy-neutral loss of heterozygosity (CN-LOH) were identified. Compared to the TCGA-UM cohort, SEA patients exhibited a lower frequency of monosomy 3 (14% vs. 53%) and a higher incidence of chromosome 1q gains (20% vs. 6%). Gains in chromosome 1q were significantly associated (*P* = 0.0289) with shorter progression-free survival (PFS). In comparison, gains in chromosome 9q were correlated with longer PFS in SEA patients, a trend not observed in the TCGA-UM cohort. BAP1 loss was detected in 20% of cases and was associated with reduced survival rates, consistent with TCGA data.

**Conclusions:**

This study highlights significant genetic differences between SEA and Western UM patients, particularly the lower incidence of monosomy 3 in SEA patients. This preliminary observation raises concerns about the reliability of using BAP1 loss alone, assessed through gene expression or immunostaining, as a sole marker for metastasis surveillance and risk stratification in Asian UM patients. These findings underscore the need for further research to determine whether additional genetic markers are required to improve prognostic accuracy in this population. Expanding molecular profiling in SEA would improve risk stratification and inform treatment strategies, while collaborative research with larger cohorts is essential to validate these findings and refine prognostic models globally.

**Supplementary Information:**

The online version contains supplementary material available at 10.1186/s40662-025-00430-8.

## Background

The incidence of uveal melanoma (UM) varies significantly across geographical regions and latitudes, ranging from less than one to over nine cases per million population annually [1]. High-incidence regions include Northern European countries such as Ireland, Norway, Netherlands and Denmark, which have reported incidence rates exceeding 8 per million annually [1]. Israel also reports a higher annual incidence rate of 6.71 per million [1]. In Southern Europe, such as Spain and Southern Italy, the incidence is lower, typically under two per million annually [2]. In contrast, the annual incidence of UM is lowest in Asian countries like South Korea (0.4 per million), Japan (0.6 per million), and Africa (0.3 per million) [1, 3], falling below one per million annually. Whilst the variation in incidence may appear to be related to latitude differences, iris pigmentation is likely to be a more significant factor since a higher incidence of UM is seen in Australia and New Zealand, located in the southern latitudes and typically have a higher population with lighter irides compared to nearby Asian countries with much lower incidence. In Asian and African countries with similar darker iris pigmentation or higher Fitzpatrick Skin Scales of IV and higher, the incidences of UM are typically lower.

Despite its rarity in Asian countries, previous studies have indicated that survival rates among Asian patients with UM are higher than those observed in Western populations [4–6], possibly due to underlying genetic and geographic differences. However, UM is often overlooked in Asia with advances in genetic prognostication being limited.

Previously, primary enucleation was the standard treatment for UM in Asia [3]. However, over the past few years, there has been a shift toward using eye-sparing techniques, such as plaque brachytherapy and endo-resection, particularly for small- to medium-sized tumours in Singapore and other parts of Southeast Asia (SEA) [3, 6]. These changes reflect the advances in managing UM in SEA, which are aimed at preserving vision.

In Singapore, data from the former Singapore Cancer Registry reported only six cases of UM out of 125 eye cancer patients between 1968 and 1995 [7]. More recent figures from the Singapore Cancer Registry between 1996 and 2016 showed an increase to 21 biopsy-confirmed cases of UM [8]. This rise in cases can be attributed to the near doubling of Singapore’s population over this period, increased life expectancy and improvements in clinical diagnostics and pathology. As a leading tertiary eye care center, we have observed an increasing number of UM cases, highlighting the ongoing challenges related to metastasis surveillance and survival prediction due to the region's absence of molecular prognostication tools.

In SEA, prognostication for UM remains primarily reliant on clinicopathological risk factors despite growing evidence from Western studies that chromosomal abnormalities such as monosomy 3 and gains in chromosome 8q are strong predictors of metastasis [1, 9–13]. Monosomy 3 is found in approximately 50% of UM cases in Western populations, while gains in 8q are present in about 40% [14]. These aberrations form the foundation of The Cancer Genome Atlas (TCGA) classification system, stratifying UM into low- and high-risk categories. Low-risk groups, including Group A (disomy 3, disomy 8) and Group B (disomy 3, 8q gain), contrast with high-risk groups, such as Group C (monosomy 3, possible 8q gain) and Group D (monosomy 3, multiple 8q gains) [15].

Gene expression profiling (GEP) has emerged as another essential tool for risk stratification in UM. The DecisionDx-UM GEP test, which classifies tumours into Class 1 (low risk) and Class 2 (high risk) based on a 15-gene panel, has gained prominence in the West [16] but remains unavailable mainly in Asia. The absence of such molecular tests in SEA highlights a critical gap in the region's UM prognostication and metastasis surveillance. In Western settings, molecular findings combined with histopathological data are used with tools like the Liverpool Uveal Melanoma Prognosticator Online III (LUMPO III) [17], the Liverpool Parsimonious Model (LPM) [18, 19], and the predicting risk of metastasis in uveal melanoma (PriMeUM) [20] to predict survival and guide clinical decision-making. The need for molecular information in Asia limits the use of these advanced resources for our patients with UM.

In recent years, studies have shown that increased preferentially expressed antigen in melanoma (PRAME) expression in tumours is associated with metastatic risk [21, 22]. Studies combining the use of PRAME mRNA expression as well as immunohistochemistry (IHC) and GEP have also been shown to improve metastatic prognostication [23–25].

Current methods, such as karyotyping and fluorescence in situ hybridization (FISH), are effective for detecting monosomy 3 and have been used in SEA. However, these techniques typically require fresh tissue, limiting their use. To overcome these limitations, newer techniques such as single-nucleotide polymorphism (SNP) analysis are gaining prominence [16, 26, 27]. As GEP is not readily available in SEA, SNP analysis offers the advantage of evaluating multiple regions across individual chromosomes. It can detect not only monosomy but also isodisomy 3, genome-wide copy number variations (CNVs), and copy-neutral loss of heterozygosity (CN-LOH) [9]. Recent studies report other cytogenetic alterations, such as losses of chromosomes 1p, 3, 6q, 8p, and 16q, and gains in 6p and 8q, also to have a prognostic role in UM [9, 13, 14, 28–31]. As these alterations are not well studied in Asian UM, applying SNP analysis can significantly advance our understanding of UM in the region.

Thus, this retrospective cohort study aims to bridge this gap by assessing chromosomal aberrations in UM tumours from our Southeast Asian (SEA) patients. We seek to correlate these cytogenetic findings with histopathological features, BAP1 IHC, and clinical outcomes, including metastasis occurrence and post-metastasis survival. Furthermore, by comparing our findings with the TCGA-Uveal Melanoma (TCGA-UM) dataset, this study explores potential differences between Asian and Western UM populations. The outcomes of this research will provide valuable insights into the pathogenesis of UM in Asia, contributing to the development of more refined prognostic tools and therapeutic strategies for this rare and challenging disease.

## Methods

### Patient sample collection and clinical examination

This research was approved by the SingHealth Centralised Institution Review Board (Reference No. 2015-2289). The formalin-fixed paraffin-embedded (FFPE) enucleated UM samples were identified using a database search of UM biopsies performed at the Singapore National Eye Center (SNEC) between 2004 and 2018. All samples were obtained before treatment and at the time of first diagnosis. De-identified FFPE sections were submitted to the Cytogenetics Laboratory in Singapore General Hospital for OncoScan™ CNV Array (Agilent, Santa Clara, California, USA) to detect chromosomal aberrations.

A retrospective review of medical records was performed to obtain information on age at the time of UM diagnosis, sex (male/female), tumour location, largest basal diameter and height, cell type, IHC BAP1 staining, and pathological TNM classification system [American Joint Commission on Cancer (AJCC) 8th edition] [32, 33].

### DNA extraction

Archived FFPE blocks were cut and fixed on glass slides, deparaffinised, and rehydrated by going through a series of xylene, ethanol, and water. Tissues were then collected in a 1.5 mL Eppendorf tube, and RNA-free genomic DNA was isolated using the QIAamp® DNA FFPE Tissue Kit and RNase A (100 mg/mL; Qiagen, Hilden, Germany) following the manufacturer’s recommendations. Isolated DNA was purified with OneStep™ PCR Inhibitor Removal Kit (Zymo Research, Irvine, California, USA) to reduce/remove pigment as per the manufacturer’s instructions. DNA concentration was determined by the Qubit™ dsDNA HA Assay Kit with Qubit 3.0 Fluorometer (Invitrogen, Carlsbad, California USA).

### Determination of chromosomal aberrations

Genome-wide copy number variations and CN-LOH were analysed using the OncoScan™ CNV Array (Applied Biosystems, Carlsbad, California, USA) according to the manufacturer’s protocol. Cel files generated automatically after chip scanning were imported into the Chromosome Analysis Suite version 4.0 (Applied Biosystems, Carlsbad, California, USA) and analysed using the FFPE Analysis NA33 workflow. The genome version of hg19 was used for annotation.

### BAP1 IHC

BAP1 IHC was conducted using the antibody clone sc-28383 from Santa Cruz Biotechnology (Dallas, TX, USA). Automated immunostaining techniques were validated in the Clinical Pathology Laboratory of the Singapore General Hospital. Lung adenocarcinoma was used as an external positive control. Positive normal endothelial cells served as internal controls.

### Data analysis

Comparisons of continuous data were performed using the unpaired *t*-test, while categorical data were analysed using the Fisher’s exact test. A *P* value of less than 0.05 indicated that the difference between the groups were statistically significant. Overall survival (OS) was calculated as the time interval from the date of diagnosis to the date of death, irrespective of the cause or the date of the last follow-up for patients still alive at the end of the study period. This approach accounts for all-cause mortality to provide a comprehensive measure of survival outcomes.

Recurrence-free survival (RFS) was defined as the time interval from the date of diagnosis to the first occurrence of disease relapse or tumour-related death, whichever occurred first. This metric captures explicitly the period during which patients remained free of recurrence or tumour-related mortality following initial diagnosis. Progression-free survival (PFS) was defined as the time interval from the initiation of first-line therapies to the date of documented disease progression, relapse, or tumour-related death, reflecting the duration of effective disease control under treatment.

As previously described [34], publicly available 80 primary UM mRNA expression and clinical data [35] were extracted from the TCGA-UM dataset and available to download from the Xena Functional Genomics Explorer of the University of California, Santa Cruz (https://xenabrowser.net/datapages/ and https://xenabrowser.net/heatmap/) [36] which is a part of the genomic data commons. The frequency of specific chromosomal aberrations, focusing on chromosomes 1, 3, 6, 8, and 9, was analysed and compared between the SEA cohort and the publicly available TCGA-UM dataset [35]. Each chromosomal alteration was evaluated independently to assess its association with PFS in both cohorts. This approach involved conducting individual statistical comparisons for each chromosomal aberration separately rather than performing a multi-variable analysis. As a result, no adjustments for multiple comparisons were applied since the study did not involve simultaneous testing of multiple variables within a single model. Instead, each comparison was treated as a standalone evaluation to highlight potential trends and associations that could inform future investigations in larger cohorts. This analysis aimed to uncover unique genetic patterns in the SEA cohort and assess their potential prognostic implications relative to chromosomal alterations observed in Western populations. Additionally, RFS and specific chromosomal aberrations in the SEA cohort were compared with OS and corresponding chromosomal aberrations in the West [37] TCGA-UM dataset [35].

## Results

Our pathology database identified 20 patients with UM enucleation specimens between 2004 and 2018. Table 1 shows patients’ demographics, tumour location, and tumour size. Table 2 summarises the tumour stage, histology, common chromosome alterations, and BAP1 IHC findings. It also includes TCGA classifications, the time to metastasis, survival post-metastasis, and follow-up time. All cytogenetic changes are presented in Table 3.

**Table 1:** Patient demographics and tumour location and size

Case No.	Ethnicity	Sex	Age	Tumour location	Tumour size (mm)^b^	Category^a^
UM1	Chinese	F	65	Choroidal, posterior to equator	4 × 1	1
**UM2**	Chinese	F	45	Choroidal, posterior to equator	19 × 12	4
UM3	Chinese	F	74	Choroidal, posterior to equator	19 × 16	4
UM4	Indonesian	M	64	Choroidal, posterior to equator	10 × 6	2
UM5	Chinese	M	75	Choroidal, equator	4 × 2	1
UM6	Chinese	M	37	Choroidal, equator with extension to ciliary body	16 × 16	4
**UM7**	Chinese	M	52	Choroidal, Posterior to equator	9 × 10	3
UM8	Eurasian	M	52	Choroidal, posterior to equator	14 × 5	2
UM9	Malay	M	30	Choroidal, posterior to equator	11 × 9	2
**UM10**	Chinese	F	72	Choroidal, posterior to equator with extension to ciliary body	22 × 12	4
UM11	Chinese	M	34	Choroidal, posterior to equator	16 × 5	3
UM12	Chinese	M	74	Anterior uvea, ciliary body	10 × 15	4
UM13	Chinese	F	66	Choroidal, posterior with extrascleral and optic nerve invasion	25 × 26	4
UM14	Malay	F	30	Choroidal, posterior to equator	20 × 13	4
UM15	Chinese	M	64	Choroidal, posterior to equator	15 × 12	3
**UM16**	Chinese	F	77	Choroidal, posterior to equator	15 × 15	4
UM17	Chinese	M	66	Choroidal, posterior to equator with extension to ciliary body	16 × 11	3
UM18	Chinese	M	35	Choroidal, anterior to equator	14 × 11	3
UM19	Malay	M	56	Choroidal, posterior to equator with extension to ciliary body	15 × 5	2
UM20	Chinese	M	77	Choroidal, anterior to equator	7 × 8	2

The text in bold highlights patients with metastasis

*UM* = uveal melanoma

^a^Category based on the American Joint Committee on Cancer (AJCC) 8th Edition

^b^Tumour size = base × height

**Table 2:** Tumour stage, histological risk factors, key genetics and time to metastasis and survival

Case No.	pT classification, stage and histology type^a^	Chr 1	Chr 3	Chr 6	Chr 8	Chr 9	TCGA subgroup^b^	BAP 1 nuclear staining	Time to metastasis (months)	Survival post metastasis (months)	Total follow-up (months)	Cause of death
UM1	pT1a, I, Spindle	–	–	–	–	–	A/B^c^	Present	NA	NA	154	Non-UM related
UM2*	pT4a, IIIA, Epithelioid	ND	**Mono-somy 3**	ND	**Gain 8**	ND	C	Loss	48 (kidney)	12	60	UM related
UM3	pT4a, IIIA, Spindle	ND	ND	*Gain 6p*	ND	Loss 9p	A	Present	NA	NA	6	Non-UM related
UM4	pT2a, IIA, Epithelioid	ND	ND	ND	ND	ND	A	Present	NA	NA	12	Non-UM related
UM5	pT1a, I, Mixed	–	–	–	–	–	A/B^c^	Present	NA	NA	6	Non-UM related
UM6	pT4b, IIIb, Mixed	ND	ND	*Gain 6p* **Loss 6q**	**Gain 8q**	Gain 9q	B	Present	NA	NA	192	Alive
UM7*	pT3a, IIB, Spindle	ND	ND	*Gain 6p* **Loss 6p**	ND	ND	A	Present	156 (lung)	9	165	UM related
UM8	pT2a, IIA, Spindle	–	–	–	–	–	A/B^c^	Present	NA	NA	156	Alive
UM9	pT2a, IIA, Spindle	Gain 1p **Loss 1p**	ND	*Gain 6p* **Loss 6p**	ND	Gain 9q Loss 9p,9q	A	Present	NA	NA	160	Alive
UM10*	pT4b, IIIB, Spindle	Gain 1q	ND	*Gain 6p* **Loss 6p**	**Loss 8p**	ND	A	Present	34 (liver)	17	51	UM related
UM11	pT3a, IIB, Mixed	ND	ND	ND	ND	Gain 9q	A	Present	NA	NA	144	Alive
UM12	pT4b, IIIB, Mixed	–	–	–	–	–	A/B^c^	Present	NA	NA	15	Non-UM related
UM13	pT4e, IIIC, Epithelioid	**Loss 1p**	**CN-LOH 3p**	*Gain 6p* **Loss 6q**	**Gain 8q**	ND	C	Hetero-genous loss	NA	NA	91	Non-UM related
UM14	pT4a, IIIA, Epithelioid	ND	ND	ND	ND	ND	A	Present	NA	NA	96	Alive
UM15	PT3a, IIB, Epithelioid, necrotic	ND	ND	ND	ND	ND	A	Present	NA	NA	94	Non-UM related
UM16*	pT4a, IIIA, Spindle	–	–	–	–	–	C/D^c^	Loss	1 (liver)	13	14	UM related
UM17	PT3b, IIIA, Mixed	Gain 1q	ND	*Gain 6p* **Loss 6q**	**Gain 8q**, 8p	ND	B	Present	NA	NA	60	Alive
UM18	pT3a, IIB, Spindle	ND	ND	*Gain 6p* **Loss 6p**	**Gain 8q**	ND	B	Present	NA	NA	84	Alive
UM19	pT2b, IIB, Mixed	–	–	–	–	–	A/B^c^	Present	NA	NA	83	Alive
UM20	pT2a, IIB Mixed	ND	**Mono-somy 3**	ND	**Gain 8q Loss 8p**	ND	C	Loss	NA	NA	72	Alive

* Indicate patients who had metastasis. Bolded text signifies poor prognosis, italicized text signifies favourable prognosis

*TCGA* = The Cancer Genome Atlas; *UM* = uveal melanoma; *ND* = not detected; *IHC* = immunohistochemistry; *NA* = not applicable; *CN-LOH* = copy-neutral loss of heterozygosity

^a^Pathology Tumour Classification and Stage grouping is based on the American Joint Committee on Cancer (AJCC) 8th Edition

^b^Based on TCGA classification for uveal melanoma

^c^TCGA classification based on BAP1 immunohistochemistry results

**Table 3:** Chromosomal aberrations detected in 14 patient samples with successful SNP-array

Case No.	Chromosomal aberration
UM2	Gain: 5, 8, 12, 17, 20, 22 Loss: 3
UM3	Gain: 5q22.1q35.3, 6p25.3p22.3, 6p22.3p21.1, 22q11.21q13.33 Loss: Xq13.1q27.3, 9p24.3p21.3, 14q32.33, 15q26.1q26.3
UM4	Gain: none Loss: none
UM6	Gain: Xp22.2, 1q31.3q44, 2q35q37.3, 5q15, 6p25.3p21.2, 6p21.2p21.1, 8q23.3q24.21, 8q24.21, 8q24.21q24.3, 8q24.3, 9q21.33, 11p11.12q11, 11q11q22.3 Loss: 6q13q27, 11q22.3q25, 16q11.2q24.3
UM7	Gain: 6p25.3p12.3, 15q21.3q26.2 Loss: 6p11.2q27
UM9	Gain: 1p35.2q44, 4p16.3q21.21, 6p25.2p21.2, 9p24.1p23, 9q31.1q34.11, 12, 13q11q12.11, 13q12.13q12.3, 13q12.3q13.1, 13q13.1q13.3, 13q14.11, 13q14.11q21.2, 14, 17q21.33q22, 17q22, 17q22q23.2, 17q23.2q25.3, 18p, 20p13p11.21, 20q11.21q13.33, 21, 22q12.3q13.33 Loss: 1p36.33p35.2, 4q21.21q35.2, 6p25.3p25.2, 6p12.3q27, 9p24.3p24.1, 9p23q31.1, 9q34.11q34.3, 13q12.11q12.13, 13q13.3q14.11, 13q21.2q34, 15, 16q, 18q, 20p13
UM10	Gain: 1q21.1q44, 6p25.3p12.1 Loss: 6p12.1q27, 8p23.3p11.21, 18p11.21q12.3, 18q21.1q21.33, 18q22.1q23
UM11	Gain: 2p25.3, 9q21.12q34.3 Loss: none
UM13	Gain: 6p25.3p21.32, 6p21.32p21.1, 7q31.33q35, 8q21.2q24.3 Loss: X, 1p36.33p12, 5q, 6q16.3q24.1, 6q24.1, 6q24.1q27, 7q35q36.3 CN-LOH: 3p21.31p21.2
UM14	Gain: none Loss: none
UM15	Gain: none Loss: none
UM17	Gain: 1q21.1q44, 2p25.3q14.3, 2q14.3q22.2, 3q23q29, 5, 6p25.3q12, 7, 8p, 8q, 12, 13q11q34, 14, 15, 16p13.3p11.1, 17p13.3p11.1, 17q12q25.3, 18p, 21, 22 Loss: 2q24.2q31.3, 6q14.1q21, 6q21, 6q21q25.3, 6q25.3q27, 10p15.3p14, 10p12.2p11.22, 11q22.3, 11q23.1q24.3 CN-LOH: 18q
UM18	Gain: 6p25.3p11.2, 8q21.11q24.3, 17q21.33q25.3 Loss: 6p11.2, 6q
UM20	Gain: 8q Loss: 3, 8p

### Ethnicity, sex, and age at presentation and follow-up

The study cohort predominantly comprised patients of Chinese ethnicity (75%), followed by Malays (15%), with smaller representations of Eurasians (5%) and Indonesian Malays (5%). This ethnic distribution is reflective of the demographic profile in the region. All the patients in our cohort had brown irides and were on the Fitzpatrick Skin Scale III to V, depending on ethnicity. The Fitzpatrick Skin Type is an objective method of classifying patients based on skin colour and sunburn sensitivity [38].

A slight male preponderance was observed; 13 male patients (65%) and 7 female patients (35%). The mean age at diagnosis was 57 ± 17 years (range: 30–77 years) with a median age of 64 (Table 1).

**Fig. 1:**
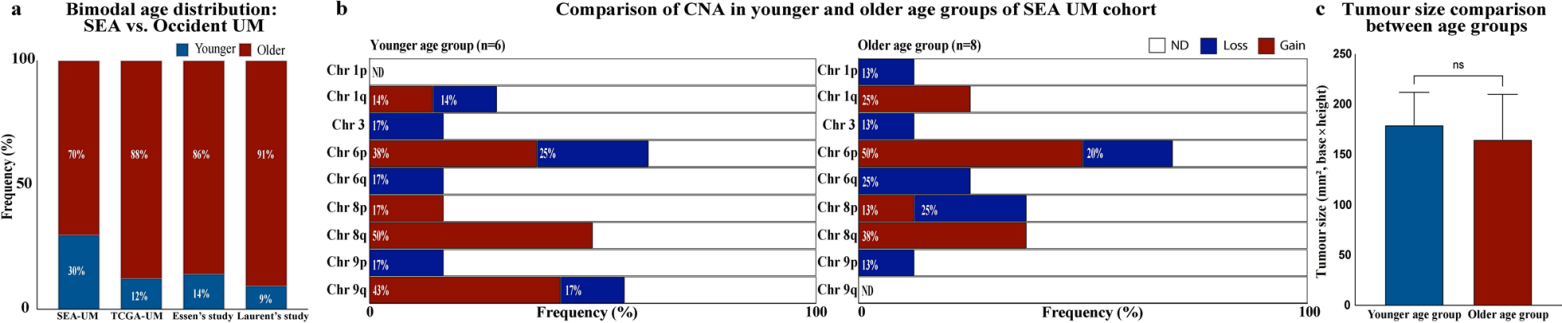
Bimodal age distribution of SEA UM cohort compared to Occident studies and comparison of chromosomal alterations and tumour volume between age groups in the SEA UM cohort. **a** Bimodal age distribution of UM: SEA vs. Occident cohorts. Comparison of the age distribution between the SEA cohort and three Occident studies: the TCGA-UM cohort, van Essen’s, and Laurent’s. The proportions of younger patients (< 45 years, blue bars) and older patients (≥ 45 years, red bars) are shown as percentages. The SEA cohort demonstrates a higher proportion of younger patients (30%) compared to the TCGA-UM cohort (12.5%), van Essen’s study (14.3%), and Laurent’s study (9.5%), highlighting potential regional differences in UM age distribution. This suggests a bimodal pattern in the SEA cohort. **b** Copy number alteration (CNA) landscape comparing the younger age group (n = 6, left panel) and the older age group (n = 8, right panel) in the SEA UM cohort. Frequencies of chromosomal gains and losses on chromosomes 1, 3, 6, 8, and 9 are shown. **c** Comparison of tumour size (mm^2^; calculated as base × height) between the younger and older age groups. There was no significant difference in tumour size between the two groups (ns). SEA, Southeast Asian; UM, uveal melanoma; TCGA, The Cancer Genome Atlas; ND, not detected

Of note, 30% (6/20) of the patients presented at a median age of 35 years (range: 30–45 years), while 70% (14/20) were diagnosed at a later median age of 66 years (range: 52–77 years), suggesting a bimodal age distribution in our SEA cohort. This finding prompted further investigation into how this age distribution compares with data from Occident studies. To further contextualise this trend, we compared our findings to three external Occident studies, including the TCGA-UM cohort [35], van Essen’s [39], and Laurent’s cohorts (Fig. 1a) [40]. The comparison highlights the higher proportion of younger patients (age less than 45 years) at 30% in our study compared to 12.5% in the TCGA-UM cohort, 14.3% in van Essen’s study, and 9.5% in Laurent’s study.

### Metastasis

Four of the 20 patients in this study developed metastasis during the follow-up period. These four patients with metastasis were of Fitzpatrick Skin Scale III and ethnically Chinese.

A slight female predominance was noted among the metastatic cases, with three females and one male affected. The median age at diagnosis for patients with metastasis was 62 years, whereas for those without metastasis, it was 64 years. Statistical analysis showed no significant difference in median age between these two groups (*P* = 0.5738), suggesting that age was not a determining factor for metastasis in this cohort. Among the four cases, two patients developed liver metastases (UM10 and UM16), which is consistent with the liver being the most common site for uveal melanoma metastasis. One patient presented with lung metastasis (UM7), and another exhibited a rare case of kidney metastasis (UM2), highlighting the variability in metastatic patterns even within a small cohort.

### Tumour location, size, and histopathological risk factors in tumours with and without metastasis

Three-quarters of the tumours (75%, 15/20) were located posterior to the equator, while a smaller proportion (20%, 4/20) involved the posterior pole and ciliary body. Only one tumour was in the ciliary body (5%, 1/20). None of the tumours were found in the iris (Table 1). Based on the TNM classification system (AJCC 8th edition), 65% of the tumours in this study were categorised as T4 (8/20) and T3 (5/20) tumours. T3 tumours were "large" tumours whilst T4 tumours were "very large" tumours. The remaining 35% of the tumours were classified as T1 (2/20) and T2 (5/20) tumours. All four of the tumours associated with metastasis were located posterior to the equator, with one extending to involve the ciliary body. Details are summarised in Table 1.

There was no significant difference in the mean basal diameter and height of the tumours between UM tumours with [mean basal diameter × height, (16.3 ± 5.6) mm × (12.3 ± 2.1) mm] and without metastasis [mean basal diameter × height, (12.1 ± 6.5) × (10.1 ± 6.4) mm, *P* > 0.05]. Age, ethnicity, pathological Tumour (pT) classification, American Joint Commission on Cancer (AJCC) staging at diagnosis, and tumour histology did not significantly affect the risk of metastasis in our patient samples (*P* > 0.05).

The histology cell types were 40% (8/20) spindle B cell tumours, 25% (5/20) epithelioid cell tumours (> 90% epithelioid component), and 35% (7/20) tumours with mixed spindle and epithelioid cell types (Table 1). In patients with metastasis, most of the tumour cell type was spindle B cell, with only one epithelioid cell type.

### Survival analysis

The median follow-up duration for the SEA UM cohort was 83.5 months (interquartile range: 60.0–151.5 months). The 5-year OS rate was 75% in all patients, while the 5-year RFS rate was 69.6%. Kaplan–Meier survival curves for OS and RFS are presented in Supplementary Fig. 1a and 1b, respectively, illustrating the survival trends over time.

The median time to metastasis was 41.0 months (interquartile range: 9.3–129.0 months). By the end of the study, 11 patients had passed on. Among these, three deaths were attributed to metastatic melanoma, while the remaining eight patients succumbed to non-melanoma-related causes (Table 2). When stratified by TNM stages, OS and RFS varied across stages, with Kaplan–Meier survival curves provided in Supplementary Figs. 1c and 1d, respectively. Although no statistically significant differences were observed between TNM stages (*P* = 0.2050 for OS; *P* = 0.1439 for RFS), trends suggest a gradual decline in survival rates with increasing tumour stage, highlighting the potential prognostic relevance of TNM staging in this cohort.

### Cytogenetic and BAP1 IHC results

Successful SNP array results were obtained from 14 samples (Table 2), with the DNA yields insufficient for the remaining six samples. A total of 78 gains, 48 losses, and 2 CN-LOH were identified in 11 samples (Table 2). The remaining three samples (UM4, UM14, and UM15) displayed a normal cytogenetic profile. There was no statistically significant difference in the age of the FFPE blocks between samples that produced successful SNP array results and those that failed due to insufficient DNA. The mean age of FFPE blocks with successful SNP results was 7.4 ± 4.9 years (range: 1–15 years), while the mean age of blocks that failed was 7.8 ± 5.0 years (range: 2–15 years), with a *P* value of 0.871, indicating that FFPE block age was not a determining factor in SNP array success. Our study also revealed that SNP array analysis from FFPE samples over ten years old was still possible with a success rate of 70% (14/20 cases). Failed cases histologically showed more necrotic areas and melanophages than viable tumours. This may have accounted for the insufficient DNA sample, as the DNA quality would be affected by necrosis, and melanin pigment would affect the DNA quality.

BAP1 IHC was performed for all 20 samples, and BAP1 nuclear staining was lost in 20% (4/20) cases. This loss in BAP1 immunoreactivity also corroborated with the monosomy or CN-LOH status on cytogenetic analysis except for one case that did not have accompanying cytogenetic results. In the four cases of metastasis, loss of BAP1 nuclear staining was only seen in 50% of cases; the remaining two cases did not have monosomy 3.

Since we did not have cytogenetic data for six of the samples, we used the BAP1 status to classify them into subgroups. Tumours with intact BAP1 staining were classified as Class A/B tumours. Of the 14 with cytogenetic data, the majority (11/14, 79%) were classified as low-risk Class A/B tumours, and only three (21%) had high-risk Class C tumours.

### Comparison of the cytogenetic data between the local Asian cohort and the TCGA-UM cohort

**Fig. 2:**
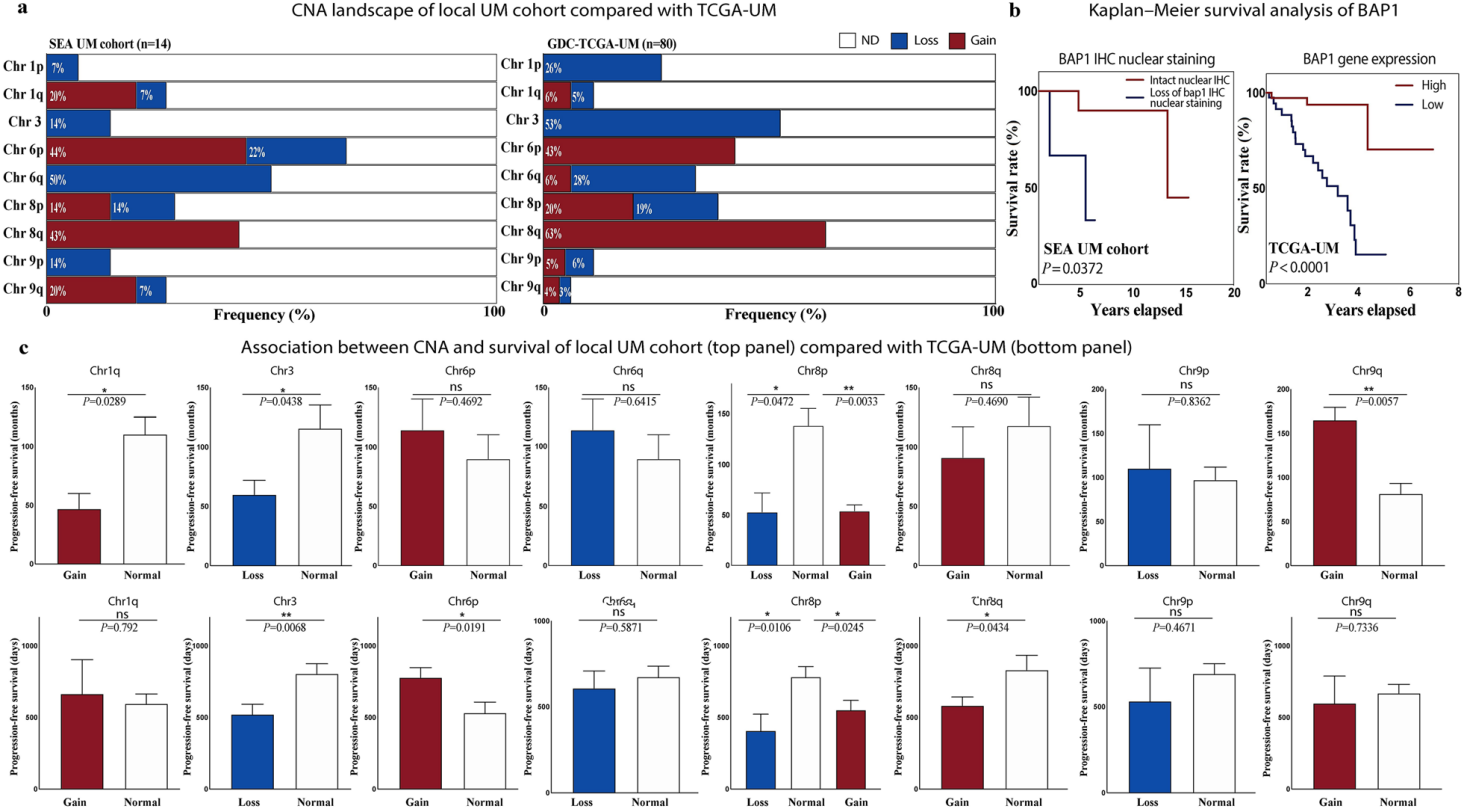
Comparison of the copy number profile between the SEA UM and TCGA-UM cohorts. **a** Copy number alteration (CNA) landscape of the local UM cohort (right panel, n = 14) compared with the TCGA-UM cohort (left panel, n = 80). Frequencies of chromosomal gains and losses on chromosomes 1, 3, 6, 8, and 9. **b** Kaplan–Meier survival curves comparing BAP1 expression levels. In the SEA UM cohort (left panel), survival is analysed based on BAP1 protein levels determined by immunohistochemistry (IHC), with red representing intact nuclear staining and blue representing loss of nuclear staining. In the TCGA-UM cohort (right panel), survival is analysed based on BAP1 gene expression levels, with “high” (red) and “low” (blue) expression categories. In both cohorts, low BAP1 expression (blue lines) is significantly associated with poorer survival compared to high expression (red lines). Statistical differences were calculated using the two-sided log-rank (Mantel-Cox) test, with time presented in years on the x-axis. **c** Association between CNAs and progression-free survival (PFS) in the SEA cohort (top panel) and the TCGA-UM cohort (bottom panel). Actual *P* values for each comparison are provided. Error bars represent the standard error of the mean (SEM). Significance levels: **P < 0.001; *P < 0.05; ns: not significant. TCGA, The Cancer Genome Atlas; SEA, Southeast Asian; UM, uveal melanoma

The frequency of previously published high-risk chromosomes 1, 3, 6, 8, and 9 are summarised in Fig. 2a [14, 30, 41, 42]. In our Asian cohort, the most frequent chromosome aberration was seen on chromosome 6 (Fig. 2a). Although chromosome 6p gains were of similar frequency with the TCGA-UM cohort, 6q losses were present almost two times more frequently in our patients (50%) than in the TCGA-UM cohort (28%, Fig. 2a). Although gains in chromosome 6p were significantly associated (*P* = 0.0191) with a longer PFS in the TCGA-UM cohort, it was not found to be statistically significant (*P* = 0.4692) in our Asian cohort.

The frequency of chromosome 9 alterations was also higher in our patients compared to the TCGA-UM cohort. Losses of chromosome 9p and gains in 9q occurred with more than two times greater frequency in our patients (14% chr 9p loss, 20% chr 9q gains, Fig. 2a) compared to the TCGA-UM group (6% chr 9p loss, 4% chr 9q gains, Fig. 2a). Gains in chromosome 9q in our Asian cohort were also significantly associated (*P* = 0.0057) with a longer PFS. However, a similar association with PFS in the TCGA-UM cohort was not detected (*P* = 0.7336) in patients with and without chromosome 9q gains (Fig. 2c).

Other differences included the higher frequency of gains in chromosome 1q and the lower frequency of chromosome 1p loss in our Asian cohort compared to the TCGA-UM cohort (Fig. 2a). In our patients, gains in chromosome 1q were significantly associated (*P* = 0.0289) with a shorter PFS (Fig. 2c). However, this was not replicated (*P* = 0.792) in the TCGA-UM cohort (Fig. 2c).

Monosomy 3 frequency was higher in the TCGA-UM cohort, present in 53% of their patients but only in 14% of our Asian cohort (Fig. 2a). In both the SEA UM and TCGA-UM cohorts, low BAP1 expression, as determined by gene expression analysis in the TCGA-UM cohort (*P* < 0.0001) and by loss of BAP antibody nuclear IHC staining in the SEA cohort (*P* = 0.0372) and was significantly associated with a reduced survival rate (Fig. 2b). Loss of chromosome 3 was also significantly associated with a shorter PFS in our Asian (*P* = 0.0438) and TCGA-UM cohorts (*P* = 0.0068) (Fig. 2c).

Chromosome aberrations involving gains and losses in 8p and gains in 8q were of similar frequency in both our Asian and TCGA-UM cohorts (Fig. 2a). In the TCGA-UM cohort, gains in chromosome 8q were significantly associated (*P* = 0.0434) with a shorter PFS. Although this trend was present in our Asian patients (*P* = 0.469), this was not statistically significant (Fig. 2c). In contrast, both gains and losses in chromosome 8p were significantly associated with a shorter PFS in our Asian (*P* = 0.0033 for 8p gain, *P* = 0.0472 for 8p loss) and TCGA-UM cohorts (*P* = 0.0245 for 8p gain, *P* = 0.0106 for 8p loss) (Fig. 2c).

### Comparison of chromosomal alterations and tumor volume between age groups in the SEA UM Cohort

Between the young and old groups, minimal differences were seen in the frequency of chr 3 losses, chr 6p, chr 8q, chr 9p (Fig. 1b).

Older patients ≥ 45 years of age showed an almost two times higher frequency of chromosome 1q gains (25%, compared to 14% in the younger patients < 45 years old) and a higher frequency of chromosome 8p losses (25%) that were not detected in the younger age group (Fig. 1b). Old patients also showed no detectable chromosome 9q changes, whilst a high number of chromosome 9q gains (43%) and a smaller proportion of 9q losses (17%) were seen in the younger age group (Fig. 1b). A slight increase in chromosome 6q losses was also present in the older age group (Fig. 1b).

In the younger patients, no detectable chromosome 1p change was seen compared to the small percentage of chr 1p losses (13%) seen in the older age group (Fig. 1b).

The comparison of tumour volume between the two age groups, calculated as the product of basal diameter and height, revealed no statistically significant difference, with both age groups displaying similar tumour sizes (Fig. 1c).

## Discussion

### Limited molecular profiling in SEA and the need for expansion

Molecular profiling in SEA remains significantly underutilised in UM, as prognostic testing in Asia relies on the IHC assessment of BAP1 loss [3, 43]. While numerous studies have confirmed the sensitivity and specificity of this IHC technique [44–46], its use is often hindered by technical issues that can lead to inaccurate assessments [45]. In our study, BAP1 IHC was used to assess its association with survival outcomes (Fig. 2), reflecting the reliance on this method in current clinical practice. However, it is essential to acknowledge that while BAP1 IHC provides critical prognostic information, it may not fully account for other genetic and molecular factors influencing UM prognosis. This underscores the urgent need to expand molecular profiling in SEA to include advanced techniques, such as next-generation sequencing or whole-genome copy number analyses, which could complement IHC and refine prognostic accuracy. Broadening the scope of molecular testing would also help address regional disparities in UM research and improve personalised treatment strategies for patients in SEA.

### Monosomy 3 and chromosome 8q in the asian context

While it is well-established that monosomy 3 and gains in chromosome 8q correlate with poor prognosis [9, 13, 29, 30, 47, 48], our study found that only monosomy 3 was significantly associated (*P* = 0.0438) with reduced PFS. The loss of BAP1 nuclear expression was also linked to shorter PFS, which matched the BAP1 gene expression in the TCGA-UM cohort. We also observed that the frequency of monosomy 3 was much lower in our Asian patients compared to the TCGA-UM cohort and other studies from the West [35, 39, 40]. Although associated with shorter PFS, the lower frequency of monosomy 3 in our cohort raises the possibility it does not play as great a role in UM metastasis and that other factors could be more critical for the disease.

Contrary to previous reports that associate 8q gains with poorer outcomes [14, 29, 30], our study found that only gains in chromosome 8p, not 8q, were linked to shorter PFS in our Asian cohort. While recent publications suggest no significant difference in survival based solely on iris colour, other studies indicate that aberrations in chromosomes 3 and 8q tend to have a more pronounced impact on patients with light-coloured irides compared to those with brown irides [48, 49]. This disparity may be attributed to differences in pigmentation and tumour biology. In our cohort, the predominance of brown irides and Fitzpatrick Skin scale III–V may partially explain why chr 8q gains were not associated with poorer outcomes. This observation aligns with the hypothesis that chromosomal aberrations such as 8q gains may be less prominent in populations with darker irides. By highlighting these differences, our findings contribute to the growing evidence that genetic and phenotypic factors, including iris colour and Fitzpatrick skin tone, may modulate the prognostic significance of specific chromosomal alterations in UM. In the TCGA cohort, patients with Fitzpatrick Skin Scale I and lighter irides were more likely to have higher grade UM compared to Fitzpatrick Skin Scale II, and III–V patients [50]. A recent study by Agrawal et al. [51] found that patients with Fitzpatrick Skin Scale III-V and brown irides had UM of larger thickness and basal diameter that corroborates with our findings. Of note, our four patients with metastasis were of the lower Fitzpatrick Skin Scale III in comparison with the rest of our cohort that ranged III–V, suggesting that skin tone is a more sensitive prognostic factor in Asians rather than iris colour, which has no grading system like the Fitzpatrick Skin Scale.

### The significance of chromosome 1q gains in Asian UM tumor progression

In our study, chromosome 1q gains, as well as chromosome 8p losses and 8p gains, were associated with shorter PFS in the SEA UM cohort. The observed frequency of 1q gains in our cohort (20%) was notably higher than that in the TCGA-UM cohort (6%) and aligns with previously reported frequencies of approximately 24% in other studies [29]. While chromosome 1q gains have been associated with poorer prognosis [29], their specific role in UM progression remains poorly understood, and research explicitly examining their impact in UM is limited. A recent study by Shain et al. on metastatic UM demonstrated that chromosome 1q gains were significantly enriched in metastatic tumours, often emerging later in disease progression following the loss of BAP1 [52]. This suggests that 1q gains represent a late event contributing to UM metastasis. In our SEA cohort, where delayed diagnosis remains a challenge, patients often present with more advanced disease, and this may contribute to the observed higher frequency of chromosome 1q gains since it occurs as a late event in the mutational timeline. These findings suggest the importance of early detection and treatment in Asian UM to mitigate the development of such late-event high-risk genetic alterations. Interestingly, in the TCGA-UM cohort, the trend of shorter PFS associated with chromosome 1q gains observed in our SEA cohort was not evident, further underscoring potential differences in genetic and clinical behaviour between Asian and Western UM populations (Fig. 2c).

The formation of isochromosomes, particularly involving chromosome 1q, may provide additional insights into the mechanisms underlying 1q gains in UM. Previous research [53] suggests that genomic instability caused by the loss of chromosome 3 can lead to the formation of isochromosomes, including those involving chromosome arms such as 1q, 6p, and 8q. This mechanism could explain the additional copies of 1q observed in our cohort, potentially in cases with concurrent monosomy 3. However, compared to the Western populations, the relatively low frequency of monosomy 3 in our SEA cohort suggests that 1q gains occur independently of chromosome 3 loss in some patients. This highlights the need for further research to explore the formation of isochromosome 1q in relation to monosomy 3 and its role in UM progression, especially in non-Western populations.

The distinct genetic landscape observed in our SEA cohort, where chromosome 1q gains were more frequent compared to the TCGA-UM cohort and were associated with shorter PFS together with the lower monosomy 3 frequency reflect differences in the underlying genetic mechanisms of metastasis between SEA and Western populations. These findings emphasize the need for the expanded study of genetic alterations in individuals with different iris colours and skin tones to further elucidate and understand such potential differences.

### Chromosome 6p loss and metastatic potential

In our study, chromosome 6p loss was the most frequently observed chromosomal aberration among patients with metastasis, present in about 50% of these cases. This finding aligns with other studies linking 6p loss to increased metastatic risk in UM [14]. In the patient with the shortest RFS and liver metastasis, chromosome 6p losses were present with other high-risk aberrations such as losses in chromosome 1q and gains in chromosome 8q, but notably without monosomy 3. This suggests that genetic aberrations without monosomy 3 such as chromosome 6p loss, together with other high-risk aberrations can be as aggressive as tumours with monosomy 3 that are known to have earlier metastasis. Whilst this raises a possibility that in Asian UM, the higher frequency of 6p losses and other non-monosomy 3 high-risk mutations can drive metastatic risk, we are cautious of drawing further conclusions due to the low statistical power of our study. Further studies with larger cohorts are needed to confirm our observation of the metastatic potential of 6p loss with other non-monosomy 3 chromosomal aberrations in Asian tumours.

### Chromosome 9q gains: a potential protective biomarker

Gains in chromosome 6p are the only aberration associated with a favourable outcome [48]. In our study, chromosome 6p gains did not demonstrate a statistically significant “protective effect” against metastasis but showed a trend toward a longer PFS. However, the study did not have sufficient power to either confirm or exclude the possibility of this protective effect, given the small sample size. Interestingly, chromosome 9q gains were significantly associated (*P* = 0.0057) with longer PFS in our cohort, suggesting a protective role. Chromosome 9q gains have not been widely reported as prognostic in UM. However, a small case series of Vietnamese UM patients with 9q gains and no metastasis was observed during a 3-year follow-up which appears to support our observation [54]. The clinical significance of this needs to be further evaluated as this was not observed in the TCGA-UM cohort.

### Mutation burden differs with age—a possible bimodal age of presentation

Our mean age of diagnosis was 57 ± 17 years, reflecting this trend of a younger age of presentation in Asian populations. This distribution aligns with findings from Manchegowda et al. who reported a generally younger mean age of presentation in Asian UM patients compared to Western populations, ranging from 42.9 to 63.5 years [3].

However, when using a cut-off age of ≥ 45 years, we observed a bimodal age distribution in our SEA cohort, with a younger subgroup presenting at a median age of 35 years (range: 30–45 years) and an older subgroup at a median age of 66 years (range: 52–77 years), that is similar to the median age reported in the West [1].

To determine if this bimodal age distribution was due to tumour volume, we compared the tumour volume between these two age groups (Fig. 1c) and found no statistically significant difference, indicating that the tumor size at presentation was similar regardless of age. In the same vein, when comparing the survival outcomes in these age subsets, there was no statistically significant difference between the younger and older age groups. However, the overall survival rate was slightly higher in the younger subgroup (83%) compared to the older group (70%). Among patients < 45 years old, only one patient experienced UM-related mortality due to kidney metastasis (Table 3), indicating a relatively favourable survival profile in the younger group.

An age-related difference in genetic landscape comprising a higher frequency of chromosome 1q gains, 8p losses, and a smaller increase in chromosome 6q and 1p losses was noted in the older age subset compared to the younger subset of patients (Fig. 1b), which may further support a possible bimodal age of presentation.

Whilst this bimodal age distribution may reflect inherent detection bias or systematic differences in tumour presentation due to our small sample size, it highlights the possibility of differences in demographic and genetic characteristics that may be unique to Asian populations. Again, future studies with larger cohorts are necessary to investigate any potential associations between age at diagnosis, genetic profiles, and tumour characteristics in UM before further definitive conclusions can be drawn.

### Tumour size and histology

Our study's tumour sizes and histological findings are similar to other reports studying Asian populations, where spindle cell histology is more commonly observed [3]. However, according to the TNM classification, 65% of the tumours in our cohort were classified as large or very large, and almost half of our tumours (9/20, 45%) were stage III tumours (Table 2, Supplementary Fig. 2), reflecting a high proportion of advanced tumours at diagnosis. This observation underscores the challenges of delayed diagnosis in Southeast Asia, where limited awareness of UM often causes patients to assume that the vision loss is attributed to more common conditions such as cataracts which in turn leads them to seek treatment when the loss in more severe. Such delays likely contribute to our study's large tumour sizes and advanced stages. Interestingly, we found no significant difference in tumour size between younger and older patients, suggesting that delayed diagnosis and its associated challenges are not confined to specific age groups but are a widespread issue across the SEA population. These findings highlight the need for increased awareness and earlier detection strategies to improve regional UM outcomes.

### Survival and metastatic rates

The 5-year OS in our cohort was 75%, within the wide range reported in Asia (53%–92%), and is only slightly higher than the 70% OS observed in the TCGA-UM cohort [35] and the 62%–70% reported in the West [3, 55]. The 5-year RFS/PFS rate in our cohort was 70%, similar to the PFS rate of 69% reported in the West [37] and 64% observed in the TCGA-UM cohort [35]. Similarly, the metastatic rate of 20% in our patients was only slightly lower than reported metastatic rates of up to 26% from USA cohorts [20, 41, 56]. In a study of a large series with more than 8000 patients by Shield et al., they found the 10-year metastatic rate for choroidal melanomas to be 25% [57]. Our observed rate of metastasis in our small cohort was also observed in a large Chinese series of 171 patients with a metastatic rate of 21% [5]. This indicates that metastatic rates are similar in the Asian and Western populations despite UM’s rarity in this region and raises a possibility that in Asia, other genetic mutations may play a more significant role in metastatic risk.

In Shield and colleagues’ report [57], they also highlighted the importance of tumour size in relation to metastatic risk. Although our SEA cohort had a high number of large and very large tumours, our data indicated no significant association between tumour size or histological subtype and metastasis, and this appears to deviate from their finding and commonly accepted knowledge. Additionally, in our small cohort, spindle cell melanomas were observed to metastasize more frequently than epithelioid melanomas. This unexpected finding may be due to our limited sample size, which reduces the statistical power to detect definitive associations and could introduce variability. These observations underscore the importance of future collaborative studies with an Asian Uveal Melanoma Registry like the TCGA cohort to allow us to study larger cohorts to validate or refute these findings and explore potential biological mechanisms that may underlie these differences.

### Limitations and future directions

Limitations of using the TCGA-UM dataset: The TCGA-UM is a series of publicly available tumours obtained from six institutions (four centers from the USA, one UK and one French Center, with more than 70% of the cases from UK and France) and unlike our series, were selected cases over 2003–2017 [35], whereas our SEA cohort is a single center consecutive case series analysis over 2004–2018.

In comparison to our cohort, none of the TCGA-UM tumours (n = 80) was classified as T1 tumours (compared 10% of our SEA cohort), and although the percentage of T2a and 2b tumours were similar 17.5% (17/80) in the TCGA-UM cohort vs. 20% (5/20) SEA cohort, most of the TCGA-UM tumours (66/80, 83%) were pT3 and larger [35], whereas only 65% (13/20) of tumours in our SEA cohort fell into this category (Supplementary Fig. 2). The higher proportion of larger tumours and the presence of metastasis at diagnosis in four patients from the TCGA-UM cohort, compared to none in our cohort, also indicate that the TCGA-UM cohort included more advanced and aggressive UM tumours. This may explain why the number of cases with metastasis during follow-up was higher in the TCGA-UM cohort (33 cases, 41%), which is double the rate observed in our SEA cohort (four cases, 20%).

Whilst the comparison with the TCGA-UM cohort provides meaningful context, there are differences in the TCGA cohort and our SEA cohort that limit direct generalizability, underscoring the need for multi-regional comparative studies across geographic regions to reflect real-world data.

Another limitation of our study is the small sample size which reflects the rarity of UM in Southeast Asia but also emphasizes the need for similar studies from other centers in this region for real-world data analysis. Despite the limited number of cases, our findings highlight key differences in genetic alterations, offering preliminary trends that may encourage similar future research. In previous studies from Asia, the relatively shorter follow-up duration may underestimate late-onset metastasis, and the absence of advanced molecular profiling techniques, such as next-generation sequencing, limits the depth of our genetic analysis. However, the use of the OncoScan™ CNV Array permits us to identify critical chromosomal alterations that influence UM progression.

Despite these limitations, this study has several strengths. It focused on a rare, understudied Asian population and provided insights into critical prognostic markers like monosomy 3 and polysomy 8 while highlighting the potential role of other chromosomal aberrations, such as chromosome 1q gains and 6q losses, in association with metastatic disease. It also reveals the need for better awareness and earlier diagnosis of UM in Southeast Asia, offering valuable clinical relevance by encouraging the use of molecular profiling in the region.

The lower incidence of monosomy 3 in our local population raises concerns about whether relying solely on BAP1 loss, assessed through gene expression or immunostaining, is sufficient for metastasis surveillance and risk stratification in Asian patients. The association of other cytogenetic abnormalities, such as chromosome 1q gains and both chromosome 8p losses and gains, with shorter PFS suggests that these mutations may play a more significant role in the metastatic process in Asians. Investigating the impact of these chromosomal aberrations in other Asian cohorts and conducting gene expression profiling will be crucial for further clarifying their roles and uncovering downstream mechanistic insights and represent our directions for future research. In addition, the role of PRAME IHC or mRNA expression may be a useful alternative in SEA and represents our future area of research. This, together with GEP analysis, may be useful for UM prognostication in SEA.

## Conclusion

In conclusion, this study provides critical insights into the genetic landscape of UM in SEA patients, highlighting potentially distinct chromosomal aberrations compared to Western populations. The lower incidence of monosomy 3 and the higher frequency of chromosome 1q gains in SEA UM patients suggest potential differences in the underlying mechanisms driving metastasis in this population. The association of chromosome 1q gains with shorter PFS emphasizes the need to further explore its role in UM progression. Additionally, identifying chromosome 9q gains as a potential protective factor underscores the complexity of the genetic landscape in SEA UM. Expanding molecular profiling and collaborating with larger cohorts will be crucial in refining these findings and improve prognostic models for UM in SEA, ultimately contributing to better risk stratification and personalized treatment strategies for this rare but significant malignancy.

## Supplementary Information

Supplementary Material 1.

## Data Availability

All data generated or analysed during this study are included in this published article and its reference.

## Abbreviations

SEA: Southeast Asian
UM: Uveal melanoma
TCGA-UM: The Cancer Genome Atlas uveal melanoma
FFPE: Formalin-fixed paraffin-embedded
CN-LOH: Copy-neutral loss of heterozygosity
GEP: Gene expression profiling
LPM: Liverpool Parsimonious Model
PriMeUM: Predicting risk of metastasis in uveal melanoma
FISH: Fluorescence in situ hybridization
SNP: Single-nucleotide polymorphism
CNVs: Copy number variations
SNEC: Singapore National Eye Center
OS: Overall survival
RFS: Recurrence-free survival
PFS: Progression-free survival
IHC: Immunohistochemistry
