# Supplementary material for: Genetic landscape of uveal melanoma in Southeast Asia: high 1q gains and unique patterns of metastasis risk
Source: Eye Vis (Lond). 2025 Apr 16;12:15. doi: 10.1186/s40662-025-00430-8 (PMC12001427; doi:10.1186/s40662-025-00430-8)

**Supplementary Figure 1. Kaplan–Meier survival analysis of the SEA UM cohort by overall survival (OS) and recurrence-free survival (RFS). a** Kaplan–Meier curve showing OS for the entire SEA UM cohort. **b** Kaplan–Meier curve showing RFS for the SEA UM cohort. **c** Kaplan–Meier curve comparing OS by TNM stages (Stage 2, Stage 3, and Stage 4). **d** Kaplan–Meier curve comparing RFS by TNM stages (Stage 2, Stage 3, and Stage 4). *P* values were calculated using the two-sided log-rank (Mantel-Cox) test. Stages are categorised based on the American Joint Commission on Cancer (AJCC) 8th Edition TNM classification. SEA, Southeast Asian; UM, uveal melanoma.

**
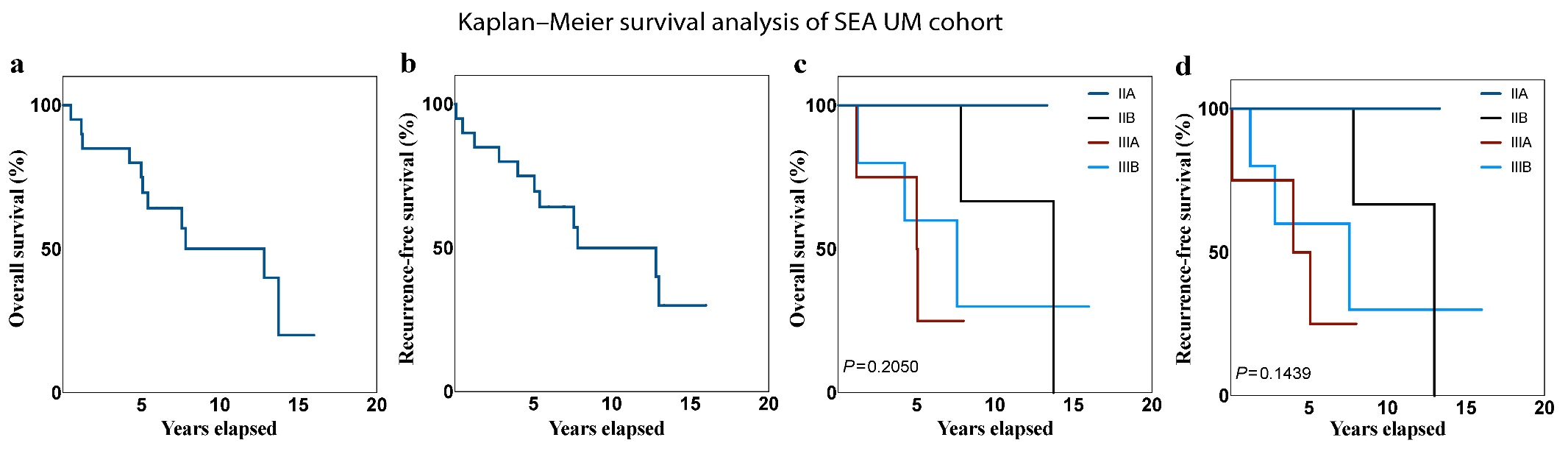
**

**Supplementary Figure 2. Comparison of AJCC pT classification and staging distribution between TCGA-UM and SEA-UM cohorts. a** Bar chart comparing the AJCC pT classification percentages between the TCGA-UM and SEA-UM cohorts. **b** Bar chart comparing the staging distribution percentages between the TCGA-UM and SEA-UM cohorts. AJCC stages and pT classifications were based on the AJCC 8th Edition TNM classification. AJCC, American Joint Commission on Cancer; TCGA, The Cancer Genome Atlas; UM, uveal melanoma; SEA, Southeast Asian


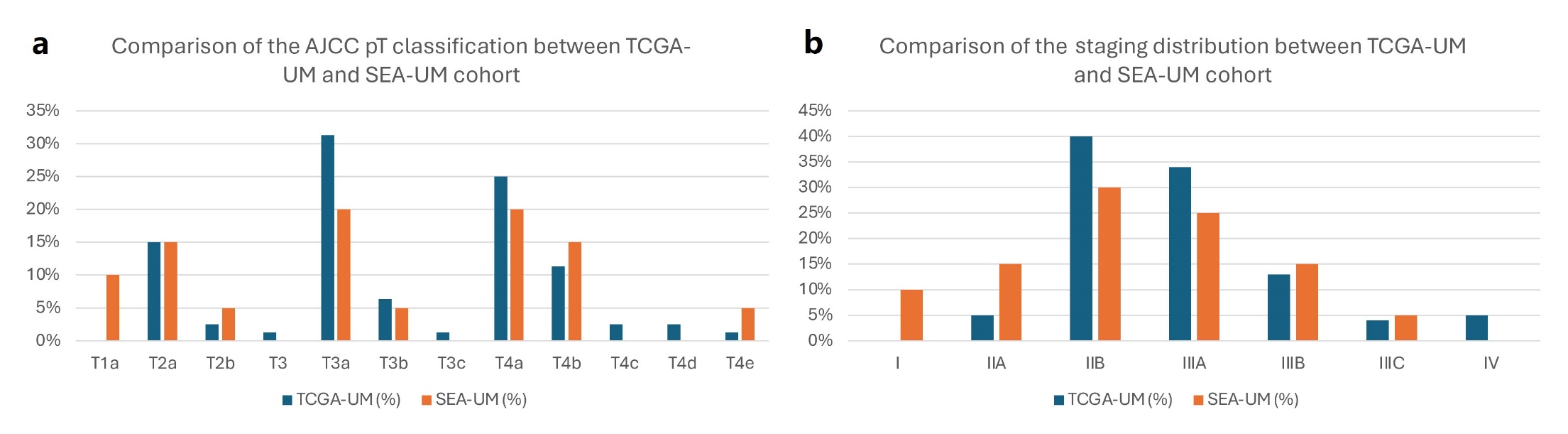

Supplement: Supplementary file 1 — Supplementary Material 1. [file 40662_2025_430_MOESM1_ESM.docx]
